# The Critical Role of Substrates in Mitigating the Power–Efficiency Trade-Off in Near-Field Thermophotovoltaics

**DOI:** 10.1021/acsami.5c17909

**Published:** 2025-11-24

**Authors:** Kartika N. Nimje, Julien Legendre, Michela F. Picardi, Alejandro W. Rodriguez, Georgia T. Papadakis

**Affiliations:** † ICFO − Institut de Ciències Fotòniques, 172281 The Barcelona Institute of Science and Technology, Mediterranean Technology Park, Av. Carl Friedrich Gauss 3, Castelldefels, Barcelona 08860, Spain; ‡ Department of Electrical and Computer Engineering, Princeton University, Princeton, New Jersey 08544, United States

**Keywords:** thermophotovoltaics, near-field radiative heat transfer, thermal photonics, substrate engineering, optimization

## Abstract

Near-field thermophotovoltaic systems can achieve ultrahigh power densities, however, this often comes at the cost of reduced efficiency. We show that this power–efficiency trade-off can be mitigated through substrate engineering. We exploit gradient-based optimization and show that thin lossless metallic films with plasma frequencies resonantly matched to the plasmonic emitter can yield high power and spectral efficiency by spectrally enhancing and confining radiative heat transfer to a narrow spectral range just above the photovoltaic bandgap. Compared to noble metals and air-bridged structures, designs deriving from such optimization yield more than an order-of-magnitude increase in radiative power density while maintaining high efficiency. Our results highlight the critical role of the substrate and the potential of substrate optimization for overcoming fundamental limitations of near-field thermophotovoltaic systems.

## Graphical abstract

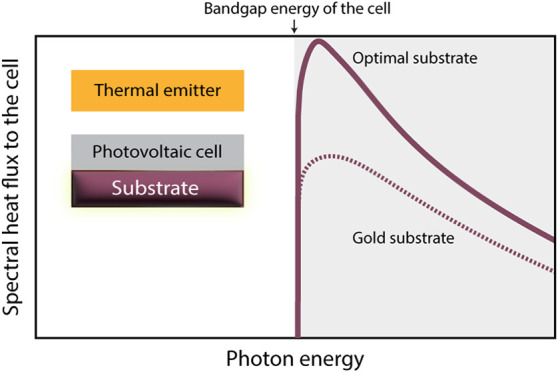

## Introduction

Thermophotovoltaic (TPV) systems convert thermal radiation into electrical power. They are increasingly being explored for applications such as industrial waste-heat recovery, space-based energy generation, and thermal energy storage.^1^ In these systems, a hot emitter radiates energy toward a photovoltaic (PV) cell across a vacuum gap. At large emitter–cell separation distances (far-field regime), this exchange is bounded by the blackbody limit. However, at submicron gaps (near-field regime), photons tunnel across the gap, enabling radiative heat transfer that surpasses far-field bounds by orders of magnitude.^2,3^ Surface polariton resonances in the emitter can further enhance this transfer, enabling near-field TPVs to achieve extremely high radiative power densities.^4–8^

Despite this promise, near-field TPVs face a longstanding challenge common to all radiative heat engines:^9^ a fundamental trade-off between power output and conversion efficiency. While increasing emitter-cell coupling boosts total radiative flux, it also amplifies spectral components that cannot be converted to electricity; photons with energies below the PV cell’s bandgap contribute to parasitic heating, while photons far above the bandgap produce carriers that rapidly thermalize. In far-field TPVs, this power-efficiency trade-off has been addressed through spectral engineering, such as selective emitters,^10,11^ multijunction PV cells,^12,13^ hot-carrier TPVs,^14^ and reflective mirrors beneath the PV cell that recycle sub-bandgap photons.^15,16^ These strategies have enabled far-field systems to achieve conversion efficiencies exceeding 40% in practice.^17^ However, such techniques cannot be directly extended to the near-field regime, which is governed by evanescent mode coupling.

Efforts to mitigate parasitic losses in near-field TPVs have primarily focused on shaping the emitter spectrum^18^ or inserting intermediate filtering layers.^19^ Yet, one key component remains overlooked: the PV cell’s substrate. In the near field, the substrate is not a passive reflector but an active participant in the radiative exchange, as it can couple directly with the emitter.^20^ The optical properties of the substrate thus critically determine whether evanescent modes contribute constructively to energy conversion or lead to parasitic losses. A poorly designed substrate can absorb a substantial portion of the heat flux, severely degrading efficiency. Recent efforts have considered air-bridge designs in near-field TPV systems,^21–25^ as these indeed partially mitigate substrate absorption. However, such solutions often fail to fully optimize the spectral selectivity in the near-field regime. In fact, what constitutes an optimal substrate for performance enhancement in near-field TPVs remains an open question.

In this work, we show that substrate engineering, even in planar geometries, offers a compelling strategy for alleviating the power–efficiency trade-off in near-field TPVs. Using fluctuational electrodynamics combined with nonlinear gradient-based optimization, we identify an unconventional yet effective solution: a thin lossless metallic (Drude) substrate with a carefully tailored plasma frequency. Such a substrate allows resonant coupling between the surface plasmon polaritons (SPPs) supported at the emitter-vacuum and cell–substrate interfaces (see Figure 1). This coupling maximizes photon absorption at frequencies near the bandgap, thereby enhancing the radiative power delivered to the cell. Simultaneously, it suppresses both below-bandgap absorption as well as thermalization by confining photon tunneling to a narrow spectral band, which enables high efficiency.

**Figure 1:**
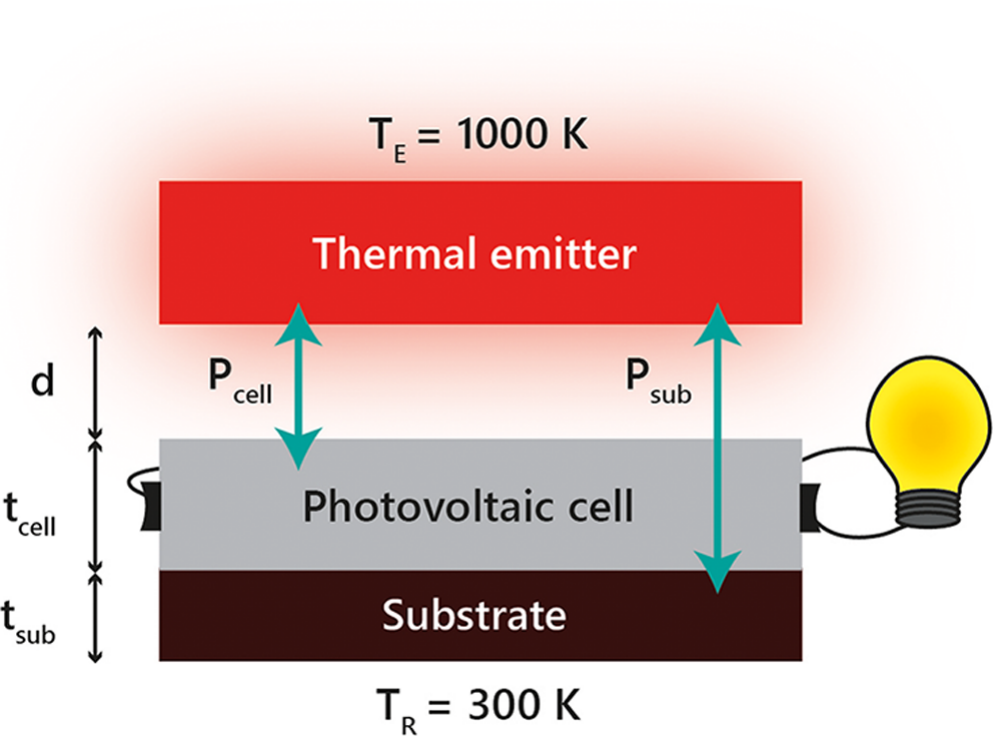
Schematic of the near-field TPV system comprising a semi-infinite thermal emitter at temperature *T*_E_ = 1000 K, a vacuum gap *d* = 10 nm, a photovoltaic cell of thickness *t*_cell_, and a substrate of thickness *t*_sub_, both held at *T*_R_ = 300 K. *P*_cell_ denotes the heat flux exchanged between the emitter and the cell, while *P*_sub_ denotes the flux exchanged between the emitter and the substrate, including radiative leakage beyond the substrate. For below-bandgap frequencies, the cell remains transparent, allowing direct radiative coupling between the emitter and the substrate.

## Theoretical Formalism

We consider a near-field TPV system consisting of a semi-infinite plasmonic emitter made of indium tin oxide (ITO), held at *T*_E_ = 1000 K, as illustrated in Figure 1. Conductive oxides such as ITO support tunable plasmonic resonances at frequencies close to the bandgap of typical TPV cells, yielding improved near-field TPV performance. The dielectric response of ITO is described by a Drude model

1 $$\varepsilon_{\mathrm{em}}\left(\omega \right)=\varepsilon_{\infty ,\mathrm{em}}\left[1-\frac{\omega_{\mathrm{em}}^{2}}{\omega \left(\omega +i\gamma_{\mathrm{em}}\right)}\right]$$

where ε_∞,em_ = 4, ω_em_ = 0.45 eV, and γ_em_ = 0.1 eV.^26^ A vacuum gap of *d* = 10 nm separates the emitter from the photovoltaic cell, ensuring operation in the near-field, which enables strong heat transfer between the emitter and cell. In particular, the power enhancement at this vacuum gap exceeds the blackbody limit by a factor of 40.^27^ To simplify the analysis, nonlocal optical effects are neglected.

The PV cell, which is placed atop a substrate (Figure 1), is maintained at a uniform temperature of *T*_R_ = 300 K. The cell has a thickness *t*_cell_, while the substrate beneath it has a thickness *t*_sub_. The dielectric response of the cell is modeled as

2 $$\varepsilon_{\mathrm{cell}}\left(\omega \right)={\left(n_{\mathrm{cell}}+i\frac{\alpha_{\mathrm{cell}}}{2k_{0}}\right)}^{2}$$

where *n*_cell_ is the refractive index, *k*_0_ = ω/c is the free-space wavenumber, and α_cell_ is the absorption coefficient. Following standard semiconductor behavior,^28^ α_cell_ exhibits a square-root dependence above the bandgap:

$$\alpha_{\mathrm{cell}}\left(\omega \right)=\{\begin{array}{ll} 0, & \omega <\omega_{g}\\ \alpha_{0,\mathrm{cell}}\sqrt{\frac{\omega -\omega_{g}}{\omega_{g}}}, & \omega \geq \omega_{g} \end{array}$$

We consider indium arsenide (InAs) as the PV cell material, with refractive index *n*_cell_ = 3.51, bandgap frequency ω_*g*_ = 0.36 eV, and absorption coefficient α_0,cell_ = 1.3 × 10^6^ m^–1^.^28^ Below the bandgap (ω < ω_*g*_), the cell is effectively transparent, exhibiting zero absorption. This is reflected in the step-like imaginary part of its dielectric function, as shown in Figure S1 of the Supporting Information (SI). This material has been considered for near-field TPVs because its bandgap closely matches the emitter spectrum for the temperatures considered.^12^ In particular, the ITO-InAs system is predicted to exhibit minimal trade-off between extracted power density and efficiency.^26,29^ To ensure strong near-field coupling between the emitter and the cell, the emitter’s plasma frequency ω_em_ is chosen such that the surface plasmon resonance of ITO, given by $\omega_{\mathrm{res},\mathrm{em}}=\omega_{\mathrm{em}}\sqrt{\varepsilon_{\infty ,\mathrm{em}}/\left(\varepsilon_{\infty ,\mathrm{em}}+1\right)}≃0.4\,\;\mathrm{eV}$ , lies slightly above the bandgap (ω_res,em_ > ω_*g*_).^26^

To understand the role of the substrate in near-field radiative heat transfer, we focus on two key photonic quantities: the radiative power above the PV cell’s bandgap (*P*_rad_) and the spectral efficiency (χ). The quantity *P*_rad_ represents the portion of the spectrum available for optoelectronic conversion in the cell, while χ captures how effectively the system manages the incoming photon spectrum, accounting for photon recycling and losses due to sub-bandgap absorption and carrier thermalization.^30^ In the limit of unity internal quantum efficiency, these quantities reduce to the conventional electrical figures of merit: the output electrical power density (*P*_el_) and the conversion efficiency (η). The quantity χ is defined as

3 $$\chi \equiv \frac{P_{\mathrm{rad}}}{P_{\mathrm{tot}}}$$

where *P*_rad_ and *P*_tot_ are given by

4 $$P_{\mathrm{rad}}=\int_{\omega_{g}}^{\infty }\int_{0}^{\infty }\hbar \omega_{g}\zeta_{\mathrm{cell}}d\beta d\omega$$

5 $$P_{\mathrm{tot}}=\int_{0}^{\infty }\int_{0}^{\infty }\hbar \omega \left(\zeta_{\mathrm{cell}}+\zeta_{\mathrm{sub}}\right)d\beta d\omega =P_{\mathrm{cell}}+P_{\mathrm{sub}}$$

*P*_tot_ is the net photonic heat flux exchanged between the emitter and the combination of PV cell and substrate (*P*_cell_ + *P*_sub_), encompassing the useful power *P*_rad_, as well as thermalization losses within the cell, parasitic absorption in the substrate, and radiative leakage beyond the substrate. In the above equation, the quantity ζ_cell/sub_ is given by

6 $$\zeta_{\mathrm{cell}/\mathrm{sub}}=\frac{\beta \omega^{2}}{4\pi^{2}c^{2}}\left[\Phi \left(\omega ,T_{E}\right)-\Phi \left(\omega ,T_{R}\right)\right]\xi_{\mathrm{cell}/\mathrm{sub}}\left(\beta ,\omega \right)$$

where ξ_cell/sub_(β, ω) denotes the photon tunneling probability^31^ at frequency ω and normalized in-plane wavevector β, and Φ (ω, *T*) = [exp (ℏω/*k*_B_ *T*) – 1]^−1^ is the Bose–Einstein occupation number. In SI, we analytically express ξ_sub_ for a semi-infinite substrate for both propagating and evanescent modes.

## Results and Discussion

We first consider conventional substrates in TPVs – gold,^100^ a perfect electric conductor (PEC), vacuum (no substrate), a gold mirror separated by a 1 μm air gap (air-bridge), and an ITO substrate. All aforementioned substrates couple differently with the ITO emitter. It is known that the thickness of the TPV cell dramatically affects device performance in the near field.^29^ In addition to influencing the cell’s optical opacity, the cell thickness determines the distance between the emitter and the substrate, and thus affects their coupling. Based on this, three regimes can be identified in Figure 2: a thin-cell regime (normalized cell thickness δ < 30, where δ = *t*_cell_/*d*) in which the emitter and substrate are close enough to exchange radiation across the entire spectrum (both above and below the bandgap); a thick-cell regime (30 < δ < 300) where the cell is optically thick to above-bandgap radiation and surface-mode coupling can only occur below the bandgap, causing the radiative power *P*_rad_ delivered to the cell to plateau; and an ultra-thick-cell regime (δ > 300) where the substrate is effectively in the far field and only propagating and frustrated modes contribute to heat transfer, causing both *P*_rad_ and the spectral efficiency χ to asymptotically saturate. These considerations indicate that substrate properties are most consequential in the thin-cell limit, where near-field coupling is strongest. In state-of-the-art near-field TPV experiments, PV cells with thicknesses on the order of a few microns are typically employed.^7,8,27^ However, for gap distances *d* < 100 nm, the dominance of high-β modes can significantly reduce the required PV cell thickness due to their low penetration depth.^32^ Our normalized thickness range thus spans both practical experimental values and exploratory ultrathin regimes.

**Figure 2:**
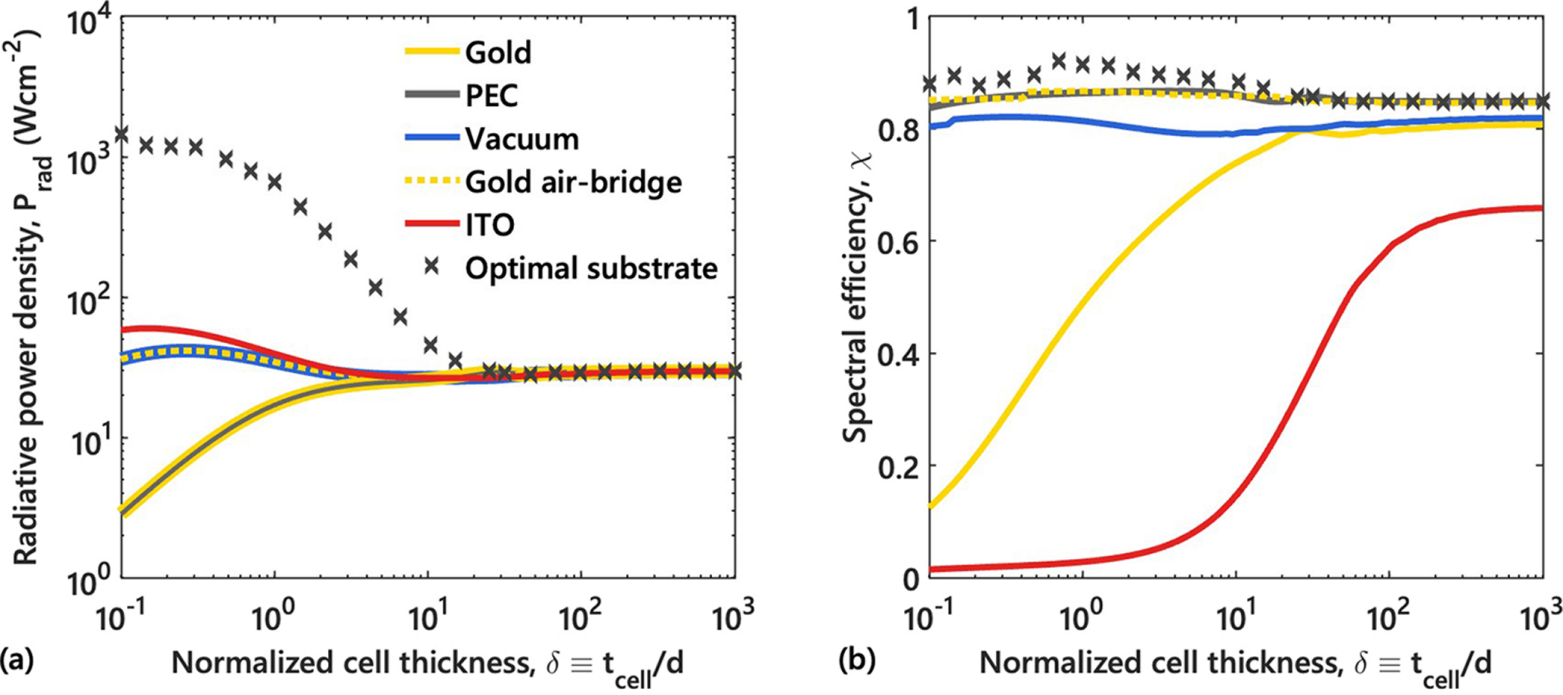
Cell thickness as a key parameter in near-field interactions: (a) Radiative power available for optoelectronic conversion, *P*_rad_ (in Wcm^–2^), and (b) spectral efficiency, χ, plotted as functions of normalized cell thickness, δ with respect to the 10 nm vacuum gap, for various substrates. Discrete markers (gray crosses) indicate optimal values obtained by jointly optimizing material parameters and substrate thickness for single-layer substrates. For completeness, the total parasitic heat flux lost in the cell and substrate (*P*_lost_) is provided in Figure S3 of the Supporting Information.

Figure 2a compares the radiative power density *P*_rad_ for the different substrates as a function of normalized cell thickness δ. For gold and PEC substrates, which do not couple strongly with the ITO emitter, *P*_rad_ is relatively low in the thin-cell regime. As the cell thickens, *P*_rad_ increases; as expected, a thicker cell simply absorbs more radiation. By contrast, with an ITO substrate or even a vacuum or air-bridge backing, *P*_rad_ can be enhanced for thinner cells due to improved near-field coupling. In fact, the highest power is obtained using a semi-infinite ITO substrate in the ultrathin limit, since the plasmonic ITO emitter can resonantly couple to an identical ITO on the backside at frequencies near the cell bandgap, greatly boosting above-bandgap radiative transfer. The vacuum and air-bridge cases also yield higher *P*_rad_ at small δ compared to gold or PEC. In these configurations, evanescent waves can tunnel between the two interfaces of the thin cell (emitter side and backside), increasing absorption in the cell even without a directly adjacent substrate.^33^ However, because vacuum and the distant gold mirror largely suppress direct emitter–substrate coupling, their *P*_rad_ in Figure 2a remains a bit lower than the ITO–ITO pairing. Overall, in the thin-cell regime we see that introducing some coupling (even in the absence of an absorbing substrate) can improve cell absorption compared to an ideal mirror or a poorly matched metal. As the cell becomes optically thick, all substrates eventually converge, making the choice of substrate largely irrelevant.

Figure 2b shows the corresponding spectral efficiency χ. Plasmonic materials like ITO and gold are detrimental to χ in the near-field regime – strong evanescent coupling enables significant radiative exchange below the bandgap (where the cell is transparent), leading to parasitic absorption in the substrate and a low spectral efficiency. This effect is most pronounced for thin cells (δ < 30), for which achieving high efficiency requires minimizing sub-bandgap coupling. Indeed, lossless substrates such as vacuum yield much higher χ at small δ by eliminating below-bandgap heat transfer due to evanescent modes. A PEC mirror represents the ideal limit, completely reflecting all photons for potential recycling, and thus maintaining a high χ. Notably, the gold air-bridge approach achieves efficiency almost on par with the PEC case. By placing the gold mirror 1 μm away, sub-bandgap photon tunneling into the substrate is prevented (similar to vacuum) while far-field (propagating) photons that would otherwise escape the system are reflected back, yielding a high χ without sacrificing much radiative power. Still, none of these conventional substrates can achieve both high power and high efficiency simultaneously.

To overcome this power-efficiency trade-off, we next turn to optimizing the substrate, employing nonlinear gradient-based local optimization tailored to the nonconvex nature of near-field radiative transfer.^34,35^ The search space spans both material parameters and substrate thicknesses, subject to physical constraints (for details on optimization, see SI). An ideal substrate should increase the radiative power delivered to the PV cell while suppressing parasitic absorption in the substrate. Accordingly, we define the objective function as

7 $$\Psi =P_{\mathrm{rad}}-P_{\mathrm{sub}}$$

To allow for a wide range of potential solutions, we began our optimization considering a general model encompassing semiconductors (eq 2), plasmonic (eq 1), and phononic (Lorentz^36^) materials as the dielectric function of the substrate, and searched for solutions that maximize Ψ (eq 7). Interestingly, for thin cells, the optimization consistently yields a Drude-like response, labeled ε_sub_(ω, ε_∞,sub_, ω_sub_, γ_sub_) henceforth. This natural selection can be understood via elimination; Lorentz-type dielectric functions with realistic phonon lifetimes^36^ are too narrow to provide sufficient above-bandgap radiative heat transfer, and doped semiconductors offer spectrally broader yet weaker evanescent coupling, making both suboptimal. For thicker cells (δ ≳ 30), multiple optima begin to emerge, with PEC becoming one viable solution. The gray crosses in Figure 2 denote the outcomes of substrate optimization performed for discrete cell thicknesses. Each marker corresponds to the optimal values obtained by jointly optimizing material parameters and substrate thickness. These optimized configurations yield substantial improvements over all conventional substrates discussed previously. For example, at δ = 1 (corresponding to a 10 nm cell), the optimal parameters are ε_∞,sub_ = 1, ω_sub_ = 1.33 eV, γ_sub_ = 0 eV, and *t*_sub_ = 15.4 nm (the variation of these parameters with cell thickness is shown in Figure S2 of the SI). These yield *P*_rad_ = 660 Wcm^–2^ and χ = 0.914. This corresponds to a 40-fold enhancement in radiative power density and a 32-fold increase in spectral efficiency compared to a gold substrate. Additionally, the optimal substrate leads to a 1.05-fold enhancement in spectral efficiency compared to a gold air-bridge substrate, and a 17-fold increase in radiative power compared to an ITO substrate. Notably, for thin cells, these values exceed those achieved with an ideal PEC reflector.

To explain the enhancement in near-field TPV performance enabled by the optimized substrate, we analyze the photon tunneling probability, ξ­(ω, β), starting from a toy model of a symmetric emitter-to-emitter system, as shown in Figure 3a. Two identical semi-infinite ITO layers form a canonical metal–insulator–metal (MIM) geometry,^37^ supporting bonding and antibonding SPP modes. For visual clarity, the ITO loss is reduced to γ′ = 5 meV. The degenerate modes exhibit maximal photon tunneling probability Em at the ITO’s SPP resonance (ω_res,em_), enabling the strongest near-field coupling.^38^ Replacing the bottom ITO layer with a plasmonic metal, termed “substrate” henceforth, of higher plasma frequency (ω_sub_ = 1.33 eV) lifts this degeneracy. As shown in Figure 3b, Em exhibits two distinct resonant branches – Em and Sub – associated with the respective SPPs of the emitter and substrate.

**Figure 3:**
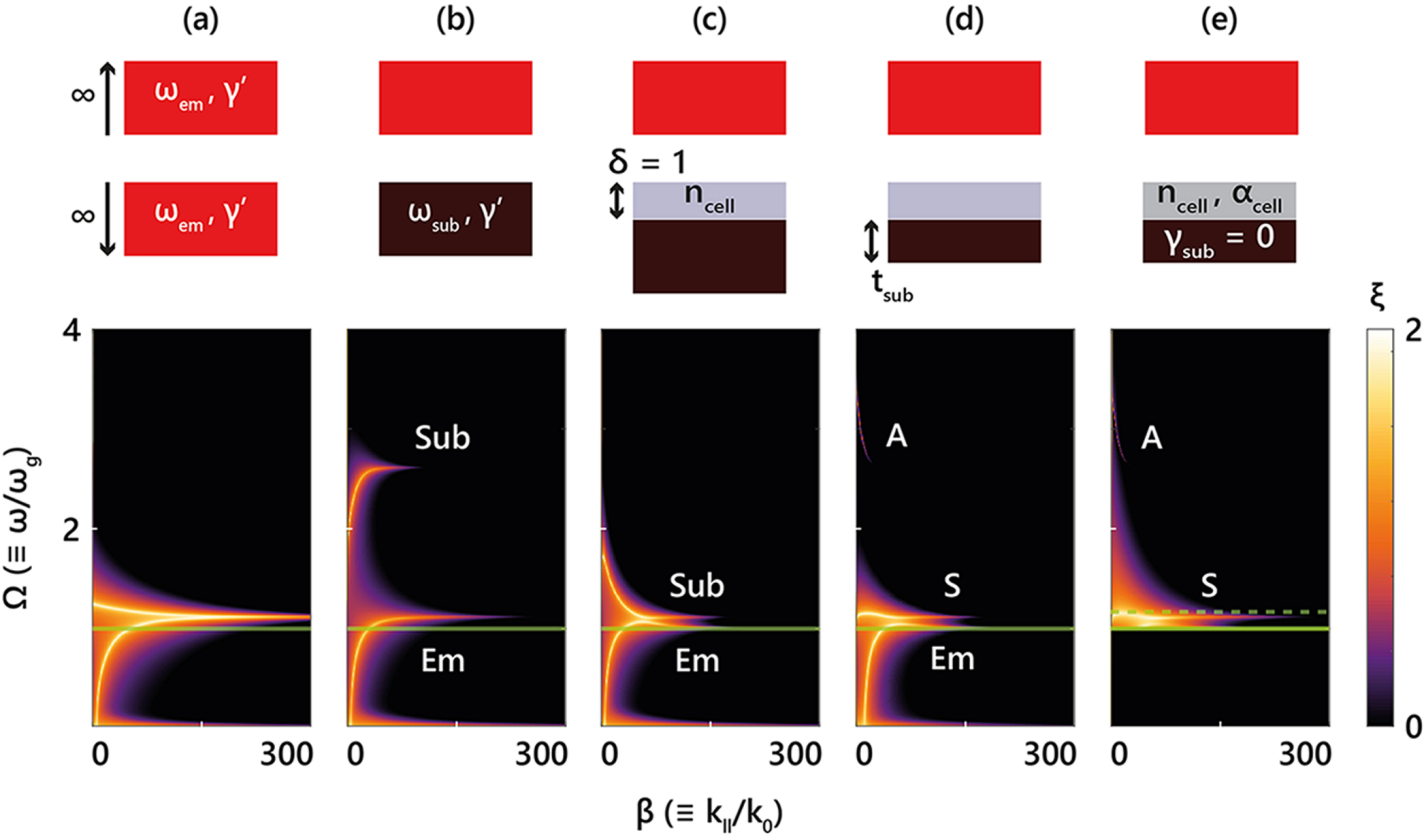
Evolution of photon tunneling: Top: schematic configurations; bottom: corresponding photon tunneling probability (ξ_cell_ + ξ_sub_) versus normalized frequency (Ω ≡ ω/ω*_g_*) and in-plane wavevector (β ≡ *k*_∥_/*k*_0_). The green solid line marks Ω = 1 (bandgap frequency). (a) Identical semi-infinite ITO layers support degenerate SPPs. (b) Increasing the plasma frequency of the substrate yields an emitter-mode (Em) and a substrate-mode (Sub). (c) Adding a 10 nm (δ = 1) dielectric redshifts the Sub mode above the bandgap, enabling spectral alignment with Em mode. (d) Reducing substrate thickness induces hybridization into symmetric (S) and antisymmetric (A) modes. (e) A lossless substrate suppresses sub-bandgap tunneling, enhancing spectral selectivity. Replacing the dielectric layer with the cell broadens the above-bandgap absorption in the cell. The green dashed line denotes the narrow bandwidth of the spectral heat flux.

When a 10 nm (δ = 1) dielectric layer is introduced above the substrate (Figure 3c), the resonance condition for the excitation of the Sub mode, now localized at the dielectric/substrate interface, shifts to lower frequencies. The Em mode is not significantly affected by the dielectric layer, since it is located at the air/emitter interface. It is ideal to redshift the Sub mode to frequencies just above the cell’s bandgap, ω_*g*_, allowing it to couple with the Em mode for maximal heat transfer. To achieve this, the refractive index of the dielectric layer plays a key role; empirically, one can write^39^

8 $$\omega_{\mathrm{sub}}\sqrt{\frac{\varepsilon_{\infty ,\mathrm{sub}}}{\varepsilon_{\infty ,\mathrm{sub}}+n_{\mathrm{cell}}^{2}}}≃\omega_{\mathrm{em}}\sqrt{\frac{\varepsilon_{\infty ,\mathrm{em}}}{\varepsilon_{\infty ,\mathrm{em}}+1}}≳\omega_{g}$$

When this condition is met, photon tunneling is spectrally concentrated near a common resonance just above ω_*g*_, maximizing useful energy transfer. The selection of a substrate with a plasma frequency larger than that of ITO warrants the proper alignment between the Em and Sub modes in the presence of the dielectric layer, which, as discussed below, represents the thin PV cell of the TPV configuration.

Next, we reduce the substrate thickness in Figure 3c to a finite, optimized value (*t*_sub_ = 15.4 nm). This leads to mode hybridization of the Sub mode into symmetric (S) and antisymmetric (A) branches, as shown in Figure 3d. The A mode, lying at a higher frequency, contributes negligibly to radiative exchange due to its spectral misalignment with the Planck distribution at *T*_E_. As a result, the above-bandgap heat transfer, primarily mediated by the S mode, becomes confined to frequencies just a few *k*_B_ *T*_E_ above the bandgap (as shown in Figure S4 of the SI), which leads to high efficiency.^40,41^ Contributions from the Em mode below the bandgap can be effectively eliminated by reducing the losses of the substrate to zero (Figure 3e), thus eliminating any heat flux at ω < ω*_g_*. This analysis is independent of the presence of loss in the cell, which merely broadens the emission above the bandgap, as shown in Figure 3e. These observations clarify why the optimal near-field TPV substrate is a lossless, thin plasmonic film with a plasma frequency higher than that of the emitter. As shown conceptually in Figure 3, this selection of the substrate maximizes emitter-substrate coupling in the presence of the cell. This, in turn, also maximizes absorption in the cell (ξ_cell_ is shown in Figure S5 of the SI), yielding large *P*_rad_ while suppressing below-bandgap absorption and thermalization losses, enabling χ higher than that for a PEC.

One may also consider the degree to which additional substrate complexity contributes to performance. We first confine the analysis to a three-layer configuration comprising the optimized plasmonic substrate, a vacuum spacer, and a far-field mirror. This design aims to recycle any residual propagating modes of radiation that leak out of the substrate. However, the performance gain is small – less than 1% – since evanescent modes dominate the heat transfer. We also examine a carefully optimized bilayer, which similarly improves the objective function (eq 7) by less than 1%. These results confirm that once the surface plasma frequency of the substrate is properly tuned, additional structural complexity yields diminishing returns. A single, lossless plasmonic layer suffices to achieve near-optimal performance.

So far, we have established a lossless Drude metal as the ideal substrate material for near-field TPV systems. Although tuning the plasma frequency of the substrate is feasible with materials such as transparent conductive oxides and nitrides, achieving near-negligible loss remains more constrained. Nonetheless, with the rapid development of plasmonic materials, it may become possible to reduce the inverse scattering time of electrons and consequently γ_sub_ through techniques such as high-temperature crystallization^42^ and chemical synthesis.^43^ To evaluate how deviations from this idealized lossless limit affect performance, we vary the damping rate γ_sub_ of the Drude model while keeping the normalized cell thickness fixed at δ = 1, reoptimizing the remaining substrate parameters for each value of γ_sub_. As shown in Figure 4, as losses increase, the objective function, Ψ, decreases sharply, from over 737 Wcm^–2^ for γ_sub_ = 0 to just 36 Wcm^–2^ for γ_sub_ = 1 eV. Beyond a threshold substrate loss of approximately γ_sub_ ≈ 0.01 eV, the optimal substrate transitions from a plasmonic metal to vacuum (variations of substrate parameters with loss and dielectric functions are provided in Figures S6 and S7 of the SI, respectively), highlighting the detrimental effect of increasing substrate losses on both *P*_rad_ and χ.

**Figure 4:**
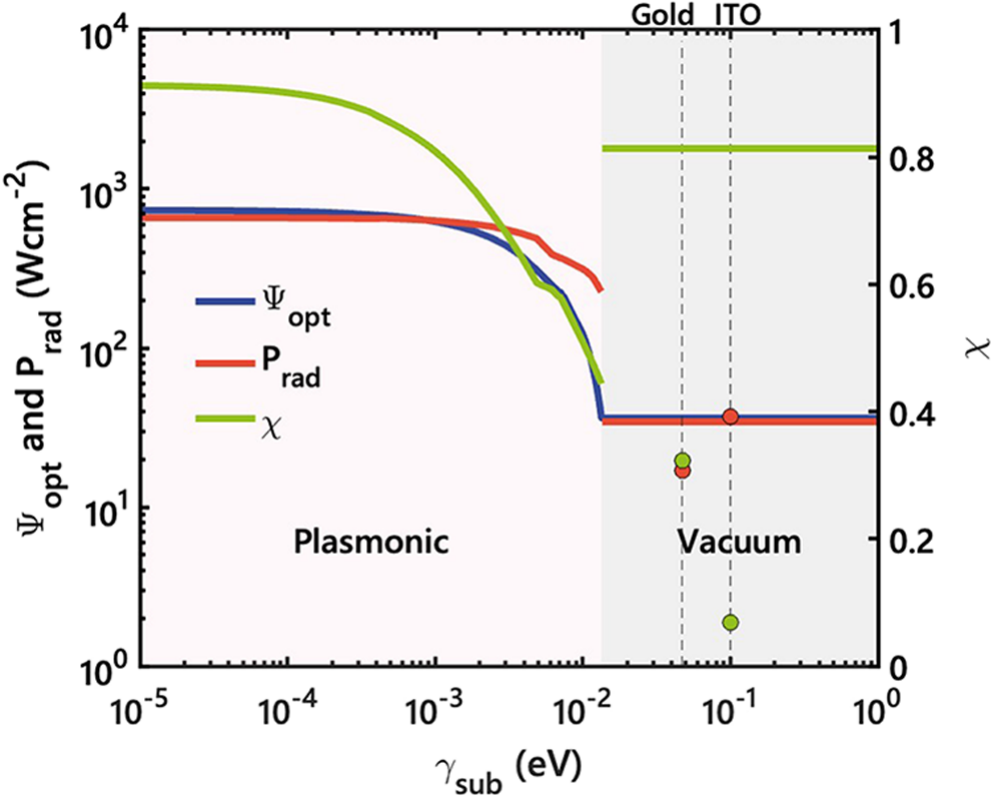
Objective function, Ψ_opt_ (blue), and corresponding radiative power density, *P*_rad_ (red), in units of Wcm^–2^, as functions of material loss γ_sub_ expressed in eV. The green curve indicates the associated spectral efficiency, χ. The pink-shaded region corresponds to regimes where the optimal substrate is plasmonic, whereas the gray-shaded region indicates that vacuum is the optimal substrate. Vertical dashed lines denote the loss parameters of gold and ITO. Colored markers highlight the values of *P*_rad_ and χ for these materials. All calculations assume a 10 nm thick cell.

## Conclusion

To summarize, we showed that careful selection of the substrate is a key and hitherto underutilized approach to alleviate the power-efficiency trade-off in near-field TPV systems. By tailoring the substrate’s optical response and thickness, we identify conditions that confine thermal emission into a narrow spectral range just above the cell’s bandgap – where photon absorption efficiently yields electron–hole pairs. The best-performing substrate is a thin lossless Drude layer, resonantly matched to the emitter’s SPP frequency. It unlocks more than an order-of-magnitude enhancement in radiative power density compared to conventional substrates, while simultaneously achieving high spectral efficiency through the suppression of sub-bandgap losses. Multilayer or mirror-backed substrates yield only marginal benefits, reinforcing the sufficiency of a single optimized layer. As material losses increase, the optimal substrate transitions from a plasmonic metal to vacuum, underscoring the importance of low-loss materials for sustaining high performance. These principles complement ongoing efforts to tailor emitter properties for enhanced selectivity and power output. Additional structural complexity such as 2D patterning (e.g., gratings or metamaterials) may yield further enhancements and/or less stringent constraints on material loss by enabling suppression of extracted radiation at undesirable (below-gap) frequencies through field cancellations and/or pseudogap effects.^44^ These results establish clear design principles for near-field spectral shaping and open new opportunities for substrate-guided optimization in TPV systems.

## Supplementary Material



## Notes

Derivation of analytical expressions; optimization details and generated data; supporting figures supporting main text results (PDF)

## References

[1] Datas, A. ; Vaillon, R. Ultra-High Temperature Thermal Energy Storage, Transfer and Conversion; Elsevier, 2021; pp 285–308.

[2] Polder D., Van Hove M. (1971). Theory of Radiative
Heat Transfer between Closely Spaced
Bodies. Phys. Rev. B.

[3] Pendry J. B. (1999). Radiative
exchange of heat between nanostructures. J.
Phys.:Condens. Matter.

[4] Narayanaswamy A., Chen G. (2003). Surface modes for near field thermophotovoltaics. Appl. Phys. Lett..

[5] Laroche M., Carminati R., Greffet J.-J. (2006). Near-field thermophotovoltaic energy
conversion. J. Appl. Phys..

[6] Shen S., Narayanaswamy A., Chen G. (2009). Surface Phonon Polaritons Mediated
Energy Transfer between Nanoscale Gaps. Nano
Lett..

[7] Mittapally R., Lee B., Zhu L., Reihani A., Lim J. W., Fan D., Forrest S. R., Reddy P., Meyhofer E. (2021). Near-field thermophotovoltaics
for efficient heat to electricity conversion at high power density. Nat. Commun..

[8] Lucchesi C., Cakiroglu D., Perez J.-P., Taliercio T., Tournié E., Chapuis P.-O., Vaillon R. (2021). Near-Field Thermophotovoltaic
Conversion with High Electrical Power Density and Cell Efficiency
above 14%. Nano Lett..

[9] Giteau M., Picardi M. F., Papadakis G. T. (2023). Thermodynamic
performance bounds
for radiative heat engines. Phys. Rev. Appl..

[10] Nefzaoui E., Drevillon J., Joulain K. (2012). Selective emitters design and optimization
for thermophotovoltaic applications. J. Appl.
Phys..

[11] Xiao C., Liu M., Yao K. (2025). Ultrabroadband and band-selective thermal meta-emitters
by machine learning. Nature.

[12] Datas A. (2015). Optimum semiconductor
bandgaps in single junction and multijunction thermophotovoltaic converters. Sol. Energy Mater. Sol. Cells.

[13] King R. R. (2025). Fundamental
advantages of multijunction thermophotovoltaic cells. Sol. Energy Mater. Sol. Cells.

[14] Nimje K. N., Giteau M., Papadakis G. T. (2024). Hot-carrier
thermophotovoltaic systems. J. Opt..

[15] Omair Z., Scranton G., Pazos-Outón L.
M., Xiao T. P., Steiner M. A., Ganapati V., Peterson P. F., Holzrichter J., Atwater H., Yablonovitch E. (2019). Ultraefficient thermophotovoltaic
power conversion by band-edge spectral filtering. Proc. Natl. Acad. Sci. U.S.A..

[16] Fan D., Burger T., McSherry S., Lee B., Lenert A., Forrest S. R. (2020). Near-perfect photon utilization in an air-bridge thermophotovoltaic
cell. Nature.

[17] LaPotin A., Schulte K. L., Steiner M. A., Buznitsky K., Kelsall C. C., Friedman D. J., Tervo E. J., France R. M., Young M. R., Rohskopf A., Verma S., Wang E. N., Henry A. (2022). Thermophotovoltaic efficiency of
40%. Nature.

[18] Karalis A., Joannopoulos J. D. (2016). Squeezing’
near-field thermal emission for ultra-efficient
high-power thermophotovoltaic conversion. Sci.
Rep.

[19] Inoue T., Watanabe K., Asano T., Noda S. (2018). Near-field thermophotovoltaic
energy conversion using an intermediate transparent substrate. Opt. Express.

[20] Bright T. J., Wang L. P., Zhang Z. M. (2014). Performance of Near-Field Thermophotovoltaic
Cells Enhanced With a Backside Reflector. J.
Heat Transfer.

[21] Inoue T., Suzuki T., Ikeda K., Asano T., Noda S. (2021). Near-field
thermophotovotaic devices with surrounding non-contact reflectors
for efficient photon recycling. Opt. Express.

[22] Feng D., Yee S. K., Zhang Z. M. (2022). Improved
performance of a near-field
thermophotovoltaic device by a back gapped reflector. Sol. Energy Mater. Sol. Cells.

[23] Lee B., Lentz R., Burger T., Roy-Layinde B., Lim J., Zhu R. M., Fan D., Lenert A., Forrest S. R. (2022). Air-Bridge
Si Thermophotovoltaic Cell with High Photon Utilization. ACS Energy Lett..

[24] Roy-Layinde B., Burger T., Fan D., Lee B., McSherry S., Forrest S. R., Lenert A. (2022). Sustaining efficiency
at elevated
power densities in InGaAs airbridge thermophotovoltaic cells. Sol. Energy Mater. Sol. Cells.

[25] Lim J., Roy-Layinde B., Liu B., Lenert A., Forrest S. R. (2023). Enhanced
Photon Utilization in Single Cavity Mode Air-Bridge Thermophotovoltaic
Cells. ACS Energy Lett..

[26] Zhao B., Chen K., Buddhiraju S., Bhatt G., Lipson M., Fan S. (2017). High-performance near-field
thermophotovoltaics for waste heat recovery. Nano Energy.

[27] Fiorino A., Zhu L., Thompson D., Mittapally R., Reddy P., Meyhofer E. (2018). Nanogap near-field
thermophotovoltaics. Nat. Nanotechnol..

[28] Ilic O., Jablan M., Joannopoulos J. D., Celanovic I., Soljačić M. (2012). Overcoming the black
body limit in
plasmonic and graphene near-field thermophotovoltaic systems. Opt. Express.

[29] Papadakis G. T., Orenstein M., Yablonovitch E., Fan S. (2021). Thermodynamics of Light
Management in Near-Field Thermophotovoltaics. Phys. Rev. Appl..

[30] Burger T., Sempere C., Roy-Layinde B., Lenert A. (2020). Present Efficiencies
and Future Opportunities in Thermophotovoltaics. Joule.

[31] Song B., Fiorino A., Meyhofer E., Reddy P. (2015). Near-field radiative
thermal transport: From theory to experiment. AIP Adv..

[100] Olmon R.
L., Slovick B., Johnson T. W., Shelton D., Oh S.-H., Boreman G. D., Raschke M. B. (2012). Optical dielectric
function of gold. Phys. Rev. B.

[32] Basu S., Zhang Z. M. (2009). Ultrasmall penetration
depth in nanoscale thermal radiation. Appl.
Phys. Lett..

[33] Francoeur M., Mengüç M. P., Vaillon R. (2008). Near-field radiative
heat transfer enhancement via surface phonon polaritons coupling in
thin films. Appl. Phys. Lett..

[34] Jin W., Messina R., Rodriguez A. W. (2017). Overcoming limits to near-field radiative
heat transfer in uniform planar media through multilayer optimization. Opt. Express.

[35] Zhang L., Miller O. D. (2020). Optimal Materials for Maximum Large-Area Near-Field
Radiative Heat Transfer. ACS Photonics.

[36] Caldwell J. D., Lindsay L., Giannini V., Vurgaftman I., Reinecke T. L., Maier S. A., Glembocki O. J. (2015). Low-loss,
infrared and terahertz nanophotonics using surface phonon polaritons. Nanophotonics.

[37] Maier, S. A. Plasmonics: Fundamentals and Applications; Springer: New York, 2007.

[38] Pascale M., Papadakis G. T. (2023). Tight Bounds and the Role of Optical
Loss in Polariton-Mediated
Near-Field Heat Transfer. Phys. Rev. Appl..

[39] Economou E. N. (1969). Surface
Plasmons in Thin Films. Phys. Rev..

[40] McSherry S., Burger T., Lenert A. (2019). Effects of
narrowband transport on
near-field and far-field thermophotonic conversion. J. Photonics Energy.

[41] Papadakis G. T., Buddhiraju S., Zhao Z., Zhao B., Fan S. (2020). Broadening
near-field emission for performance enhancement in thermophotovoltaics. Nano Lett..

[42] Lotkov E. S., Baburin A. S., Ryzhikov I. A., Sorokina O. S., Ivanov A. I., Zverev A. V., Ryzhkov V. V., Bykov I. V., Baryshev A. V., Panfilov Y. V., Rodionov I. A. (2022). ITO film stack engineering
for low-loss
silicon optical modulators. Sci. Rep.

[43] Liu L., Krasavin A. V., Zheng J., Tong Y., Wang P., Wu X., Hecht B., Pan C., Li J., Li L., Guo X., Zayats A. V., Tong L. (2022). Atomically Smooth Single-Crystalline
Platform for Low-Loss Plasmonic Nanocavities. Nano Lett..

[44] Joannopoulos, J. D. ; Winn, J. N. ; Johnson, S. G. Photonic Crystals: Molding the Flow of Light, Second ed.; Princeton University Press: Princeton, NJ, 2011.

