# Supplementary material for: The Critical Role of Substrates in Mitigating the Power–Efficiency Trade-Off in Near-Field Thermophotovoltaics
Source: ACS Appl Mater Interfaces. 2025 Nov 24;17(49):66641–7. doi: 10.1021/acsami.5c17909 (PMC12874213; doi:10.1021/acsami.5c17909)
Supplement: Supplementary file 1 [file am5c17909_si_001.pdf]

Supporting Information

The Critical Role of Substrates in Mitigating the Power–Efficiency Trade-Off in Near-Field Thermophotovoltaics

Kartika N. Nimje,[†] Julien Legendre,[†] Michela F. Picardi,[†] Alejandro W. Rodriguez,[‡] and Georgia T. Papadakis[*,†]

†*ICFO – Institut de Ciències Fotòniques, The Barcelona Institute of Science and Technology, Mediterranean Technology Park, Av. Carl Friedrich Gauss 3, Castelldefels (Barcelona) 08860, Spain*

‡*Department of Electrical and Computer Engineering, Princeton University, Princeton, NJ 08544, USA*

E-mail: georgia.papadakis@icfo.eu

Photon tunneling probability: analytical formalism

We employ a formalism based on Green's dyadic tensor[1–4] to derive an analytical expression for the photon tunneling probability between the emitter and substrate, ξ_{sub}, in the layered structure consisting of a semi-infinite emitter - vacuum - cell - semi-infinite substrate, as shown in the Fig. 1 of the main text. For notational convenience, we denote the dielectric functions of the emitter, vacuum, cell, and substrate as $\varepsilon_1, \varepsilon_2, \varepsilon_3$, and ε_4, respectively, corresponding to $\varepsilon_{\text{em}}, 1, \varepsilon_{\text{cell}}$, and ε_{sub} in the main text. The photon tunneling probability for TM

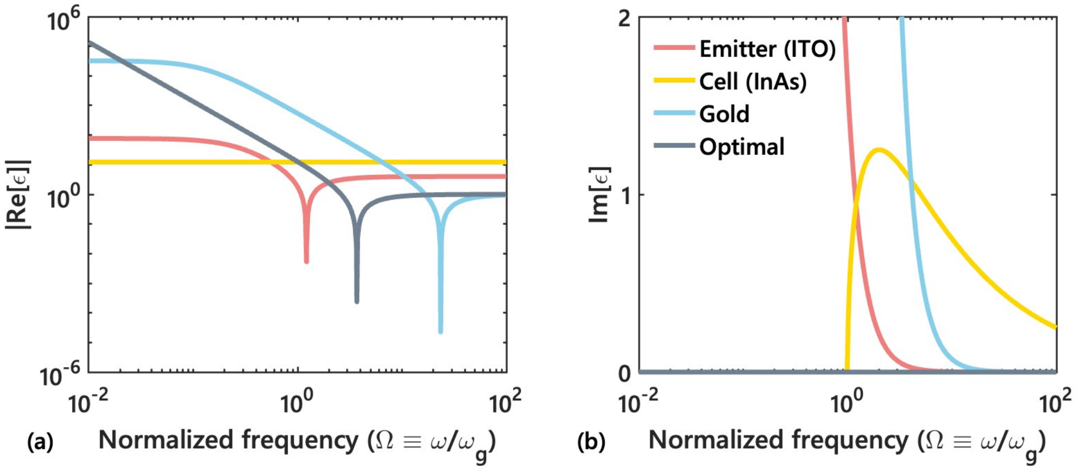

Figure S1: (a) Real and (b) imaginary parts of the permittivity of the emitter (ITO), PV cell (InAs), gold substrate, and optimal single-layer substrate for a 10 nm thick cell ($\delta = 1$ in Fig. 2 of the main text), plotted as functions of normalized frequency ($\Omega \equiv \omega/\omega_g$), with ω_g denoting the bandgap frequency of the cell.

and TE polarizations is then given by:

$$\xi_{\text{sub}}^{\text{TM}} = \frac{1}{|\Gamma_1|^2} \frac{\Re(\varepsilon_1 \Gamma_1^*) \Re(\varepsilon_4 \Gamma_4^*)}{|\varepsilon_1|\,|\varepsilon_4|} \left|\tau^{\text{TM}}\right|^2 = g_{\text{TM}}\left(\varepsilon_1, \varepsilon_4\right) \left|\tau^{\text{TM}}\right|^2, \tag{S1}$$

$$\xi_{\text{sub}}^{\text{TE}} = \frac{1}{|\Gamma_1|^2} \Re(\Gamma_1) \Re(\Gamma_4) \left|\tau^{\text{TE}}\right|^2 = g_{\text{TE}}\left(\varepsilon_1, \varepsilon_4\right) \left|\tau^{\text{TE}}\right|^2, \tag{S2}$$

where $g_{\text{TM}}(\varepsilon_1, \varepsilon_4)$ and $g_{\text{TE}}(\varepsilon_1, \varepsilon_4)$ are material-dependent prefactors. The term $\Gamma_i = k_0\sqrt{\varepsilon_i - \beta^2}$ represents the out-of-plane wave vector in the i-th medium where β is the in-plane wave vector in terms of $k_0 = \omega/c$. The term τ^q denotes the transmission coefficient, for polarization state $q = \{\text{TM, TE}\}$, which accounts for multiple reflections within the layered structure and is expressed as:

$$\tau^q = \frac{t_{12}^q t_{23}^q t_{34}^q e^{i\beta k_0 d(\delta+1)}}{e^{2i\beta k_0 d(\delta+1)} + r_{12}^q r_{23}^q e^{2i\beta k_0 d\delta} + r_{23}^q r_{34}^q e^{2i\beta k_0 d} + r_{12}^q r_{34}^q}. \tag{S3}$$

Here, r_{ij}^q and t_{ij}^q represent the Fresnel reflection and transmission coefficients for the i-j interface.

To examine the role of the cell in photon tunneling, we compare the full structure to a

simplified system without the cell. Setting $\delta = 0$ (that is, removing the cell) modifies the transmission and reflection coefficients as follows: $t_{23}^q = 1, r_{23}^q = 0$, and $t_{34}^q \to t_{24}^q, r_{34}^q \to r_{24}^q$. Consequently, the photon tunneling probability is written as $\xi_{\text{sub},0}^q$ analogous to Eqs. (S1)-(S2), with the transmission factor modified as $\tau^p \to \tau_0^q$, given by:

$$\tau_0^p = \frac{t_{12}^q t_{24}^q e^{-i\beta k_0 d}}{1 + r_{12}^q r_{24}^q e^{-2i\beta k_0 d}}. \tag{S4}$$

As g_{TM} and g_{TE} remain unchanged, comparing ξ_{sub}^q with $\xi_{\text{sub},0}^q$ leads to:

$$\xi_{\text{sub}}^q = \xi_{\text{sub},0}^q \left| \frac{\tau^q}{\tau_0^q} \right|^2, \tag{S5}$$

where $\xi_{\text{sub},0}^q$ can be described by a well-known analytical result for two semi-infinite planar bodies.[5] Essentially, by fine-tuning the reflective and transmissive properties of the material, we can directly influence photon tunneling probabilities. This result also reveals the modulation of photon tunneling by the cell and quantifies how the presence of the cell alters photon tunneling.

Optimization details

The optimization is carried out over both material and geometric parameters of the substrate. Specifically, we vary the plasma frequency ω_{sub}, damping rate γ_{sub}, background permittivity $\varepsilon_{\infty,\text{sub}}$, and thickness t_{sub}. Design variables are bounded within physically realistic ranges: $\omega_{\text{sub}} \in [0, 10]$ eV, $\gamma_{\text{sub}} \in [0, 10]$ eV, $\varepsilon_{\infty,\text{sub}} \in [1, 400]$, and $t_{\text{sub}} \in [1, 10^4]$ nm. We implement the optimization in Python using the `NLOPT` library with the `LBFGS` algorithm, and gradients are obtained via automatic differentiation. To ensure robustness against local minima, multiple initial guesses were tested, particularly for the material dispersion parameters. The optimization is terminated once the change in objective value falls below 10^{-5}. This optimization framework is used to generate the results shown in Fig. 2 and 4 of the main text. The op-

timal substrate parameters identified in this procedure yield narrowband photon tunneling concentrated just above the cell bandgap, leading to simultaneous enhancement in radiative power and spectral efficiency.

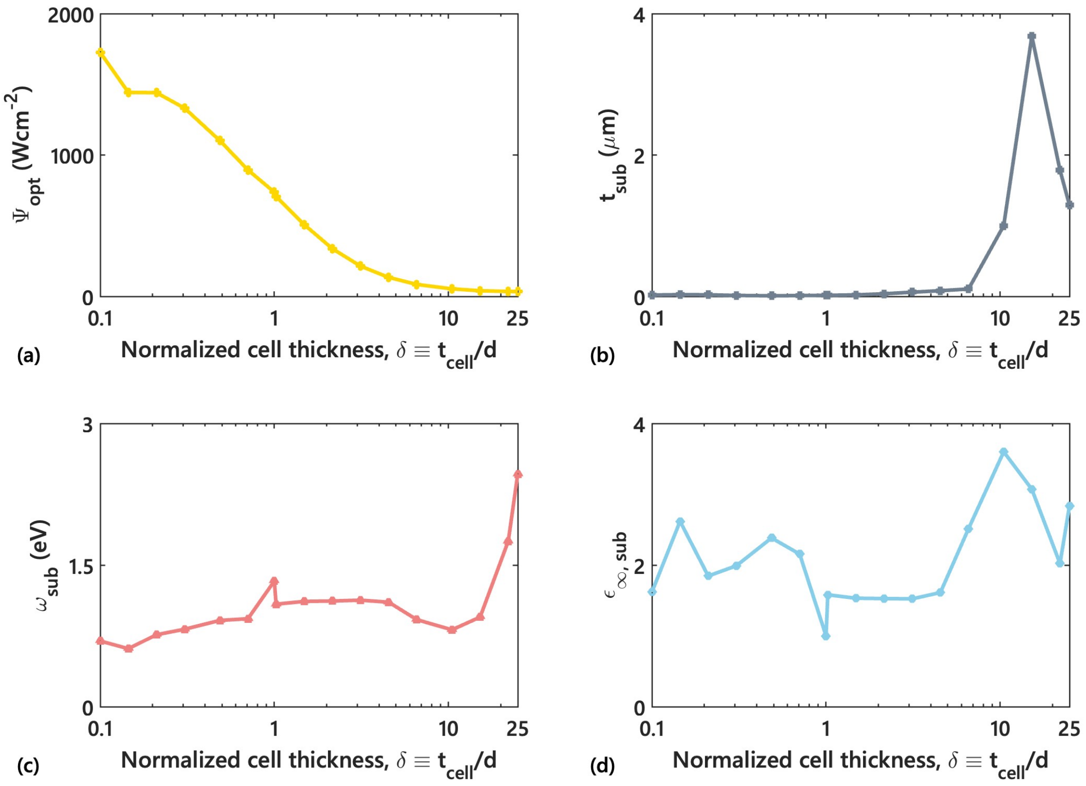

Figure S2: Optimal (a) objective function Ψ_{opt} (in Wcm^{-2}), (b) layer thickness t_{sub} (in μm), (c) plasma frequency ω_{sub} (in eV), and (d) background permittivity $\varepsilon_{\infty,\mathrm{sub}}$ of the substrate, plotted as functions of the normalized cell thickness δ with respect to the 10 nm vacuum gap. These results correspond to the optimal single-layer substrate configurations marked by gray crosses in Fig. 2 of the main text. Beyond $\delta \approx 25$, multiple optima emerge, including a perfect electric conductor (PEC) as one possible solution.

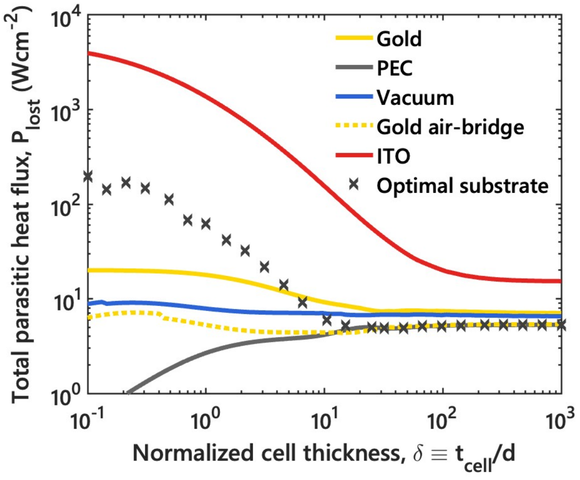

Figure S3: Total parasitic heat flux lost in the cell and substrate ($P_{\mathrm{lost}} = P_{\mathrm{ph}} - P_{\mathrm{rad}}$) in Wcm^{-2} as a function of normalized cell thickness (δ) for various substrates. This plot complements Fig. 2 in the main text. In the ultrathin limit, P_{lost} is primarily determined by evanescent coupling between the emitter and the substrate, leading to significant heat transfer. As the cell thickness increases, evanescent interactions weaken, causing P_{lost} to gradually decrease until it plateaus.

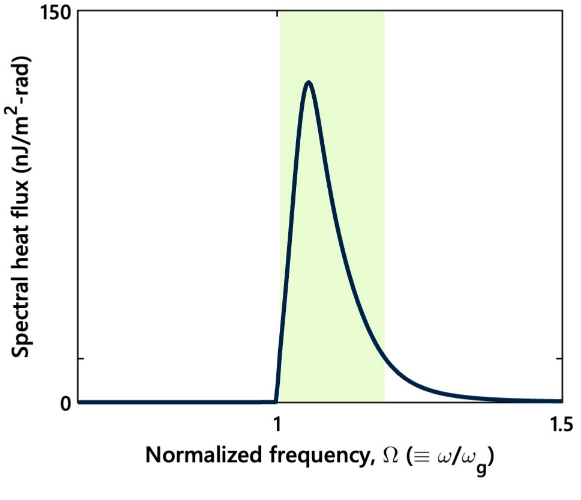

Figure S4: Spectral heat flux as a function of normalized frequency ($\Omega \equiv \omega/\omega_g$), illustrating narrowband radiative transfer dominated by a single spectral peak corresponding to the symmetric (S) mode. The shaded region denotes the frequency range above the cell bandgap that captures over 90% of the total radiated power. The spectral bandwidth, defined as the full width at which the heat flux drops to $1/e^2$ of its maximum value, is 67.8 meV, indicating strong emitter–substrate coupling and efficient, spectrally selective heat transfer.

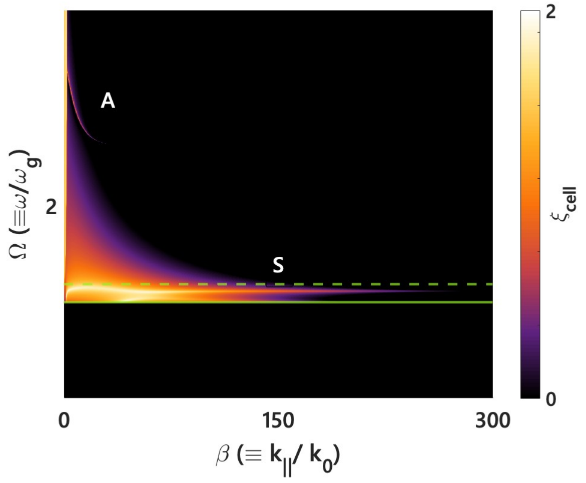

Figure S5: Photon tunneling probability between the emitter and cell ($\xi_{\rm cell}$) versus normalized frequency ($\Omega = \omega/\omega_{\rm g}$) and in-plane wavevector ($\beta = k_{\parallel}/k_0$). The green solid line marks $\Omega = 1$, corresponding to the cell's bandgap frequency. Strong emitter–substrate coupling enhances absorption in the cell, thereby increasing radiative power. This coupling produces a narrowband tunneling channel above the bandgap, dominated by the symmetric (S) mode, while sub-bandgap tunneling is suppressed by the lossless substrate. As explained in the main text, the antisymmetric (A) mode contributes negligibly to the spectral heat flux to the cell. As a result, spectral efficiency is improved. The green dashed line indicates the narrow spectral width of the radiative heat flux.

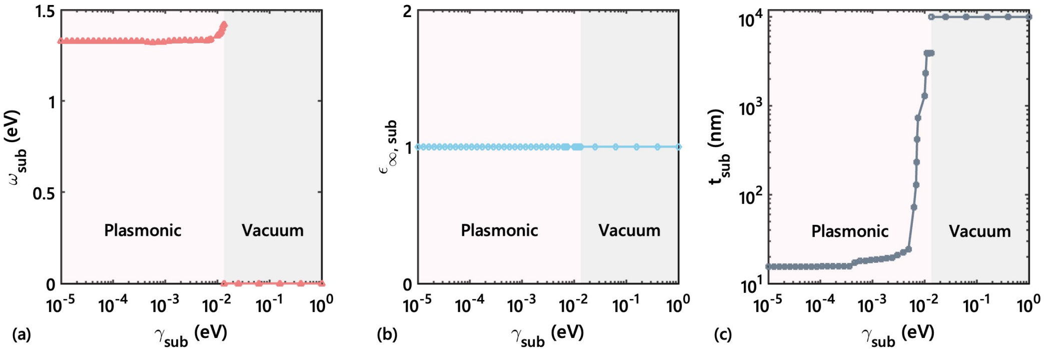

Figure S6: Optimal (a) plasma frequency $\omega_{\rm sub}$ (in eV), (b) background permittivity $\varepsilon_{\infty,\rm sub}$, and (c) thickness (in nm) of the substrate as functions of material loss $\gamma_{\rm sub}$ (in eV), corresponding to the optimal configurations shown in Fig. 4 of the main text. The pink-shaded region denotes the plasmonic regime, where the optimal substrate exhibits metallic behavior, while the gray-shaded region indicates that vacuum is the optimal choice.

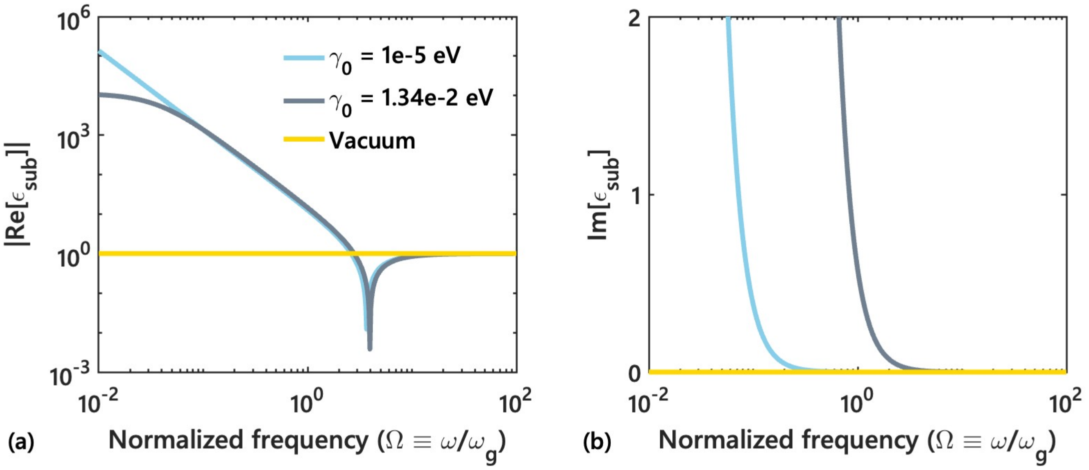

Figure S7: (a) Real and (b) imaginary parts of the permittivity $\varepsilon_{\mathrm{sub}}$ of the optimal substrate as a function of normalized frequency $\Omega \equiv \omega/\omega_g$, where ω_{g} is the bandgap frequency of the cell, for two material loss values within the plasmonic regime: $\gamma_0 = 10^{-5}$ eV (blue) and $\gamma_0 = 1.34 \times 10^{-2}$ eV (gray), corresponding to the first and last points in the plasmonic region of Fig. 4 (main text). The permittivity of vacuum is shown for comparison (yellow).

References

(1) Mulet, J.-P. Modélisation du rayonnement thermique par une approche électromagnétique. Rôle des ondes de surface dans le transfert d'énergie aux courtes échelles et dans les forces de Casimir.

(2) Francoeur, M.; Pinar Mengüç, M.; Vaillon, R. Solution of near-field thermal radiation in one-dimensional layered media using dyadic Green's functions and the scattering matrix method. *Journal of Quantitative Spectroscopy and Radiative Transfer* **2009**, *110*, 2002–2018.

(3) Blandre, E. Rayonnement thermique à l'échelle nanométrique: Effets de champ proche et d'interférences dans les structures multicouches et sur les performances électriques des cellules thermophotovoltaïques.

(4) Legendre, J. Theoretical and numerical analysis of near-field thermophotonic energy harvesters.

(5) Song, B.; Fiorino, A.; Meyhofer, E.; Reddy, P. Near-field radiative thermal transport: From theory to experiment. *AIP Advances* **2015**, *5*, 053503.
